# Enhancing seizure control in ultra-refractory postencephalitic epilepsies using multinodal network neuromodulation

**DOI:** 10.1016/j.ebr.2025.100755

**Published:** 2025-02-17

**Authors:** Subhiksha Srinivasan, Surya Suresh, Ganne Chaitanya, Manoj Saranathan, Nitin Tandon, Sandipan Pati

**Affiliations:** a Texas Institute of Restorative Neurotechnologies, Department of Neurology, The University of Texas Health Science Center at Houston, TX, USA; b Department of Radiology, University of Massachusetts Chan Medical School, Boston, USA; c Texas Comprehensive Epilepsy Program, Department of Neurosurgery, The University of Texas Health Science Center at Houston, TX, USA

**Keywords:** Encephalitis, RNS, DBS, VNS

## Abstract

This case series reports the formidable challenge posed by postencephalitic epilepsies, characterized by frequent drug-resistant seizures and neuropsychiatric and cognitive comorbidities. Polypharmacy is frequently required, and surgical resection may not be feasible due to multifocality. Neuromodulation therapies, including Deep Brain Stimulation (DBS) and Responsive Neurostimulation (RNS), offer a potential lifeline. In this case series, we shed light on the intricate landscape of seizure management and neuropsychiatric comorbidities in five individuals with frequent seizures (often weekly) and ultra-refractory epilepsy (defined as resistance to more than six different antiseizure medications, including failed epilepsy surgery) following catastrophic encephalitis. Four out of five patients achieved at least 50% reduction in seizure frequency following multimodal neuromodulation interventions. Moreover, we underscore the pivotal role of RNS electrocorticography (ECoG) in monitoring the epileptiform burden to guide therapy. Postencephalitic patients often present with a complex interplay of epileptic and nonepileptic (including neuropsychiatric) events, necessitating distinct therapeutic approaches. RNS ECoG emerges as a critical tool for differentiation and tailored therapy. While our findings highlight the potential effectiveness of neuromodulation in managing postencephalitic epilepsy, further research is needed to identify predictors of treatment response and explore the application of these therapies in chronic epilepsy caused by encephalitis. Overall, neuromodulation offers hope for improving these patients’ quality of life.

## Highlights

• Multinodal neuromodulation pioneering the future of brain health.
• DBS, VNS, RNS cutting seizures down in postencephalitic epilepsy.
• RNS ECoG unmasking the mystery between epileptic and non-epileptic events.

## 1 Introduction

Survivors of catastrophic encephalitis often encounter a significant challenge: the development of multifocal epilepsies resistant to multiple antiseizure medications (ASMs) [1]. This challenge is exacerbated by the presence of multifocal brain structural abnormalities, progressive atrophy, and significant neuropsychiatric and cognitive comorbidities [2]. Furthermore, the common occurrence of adverse effects due to polypharmacy compounds the difficulties faced by these patients. Surgical resection is often impractical due to multifocal disease presentation, however neuromodulation may be a promising option [3], [4], [5].

This case series explores the intricate terrain of managing seizures and neuropsychiatric comorbidities using intracranial neuromodulation, offering potential relief for patients with “ultra-refractory” epilepsy post-catastrophic encephalitis. For clarity, “ultra-refractory epilepsy” signifies the failure of more than six different ASMs and, in addition, unsuccessful epilepsy surgery [6]. These patients often face less favorable treatment outcomes and a heightened risk of Sudden Unexpected Death in Epilepsy (SUDEP) [7].

This report focuses on five individuals contending with frequent seizures and medically refractory epilepsy as a consequence of encephalitis, with our primary objective being to elucidate the outcomes of neuromodulation interventions in these challenging cases.

## 2 Methods

A single-institution retrospective study was conducted at Houston's University of Texas Health Science Center. Ultra-refractory postencephalitic epilepsy patients receiving treatment with Deep Brain Stimulation (DBS), Responsive Neurostimulation (RNS), and Vagus Nerve Stimulation (VNS) were included in the study. Demographics, case history, imaging, and findings from RNS and DBS recordings were reviewed and reported. Seizure frequency was obtained from patient documentation of seizure diaries as well as review of RNS Patient Data Management System (PDMS) data. The decision of target location of the various thalamic nuclei was based on the Epilepsy Conference consensus after Phase 1 and Phase 2 evaluations. The Institutional Review Board approved the retrospective chart review.

## 3 Results

The case series included five patients, two females and three males, between the ages of 13 and 46. All patients were diagnosed with ultra-refractory postencephalitic epilepsy and received treatment with intracranial neuromodulation. Four out of five patients (80 %) experienced a reduction of at least 50 % in seizure frequency. Their cases are summarized in Table 1, with additional details provided below.

Case #1 (combined RNS and DBS neuromodulation)

**Table 1:** Patient clinical characteristics with details of stimulation site and parameters.

**Patient ID**	**1**	**2**	**3**	**4**	**5**
**Age**^a^**/Sex**	26/M	37/M	48/F	23/F	11/M
**Seizure type**	GTCS	GTCS	FIAS, GTCS	FIAS, GTCS	FIAS, GTCS
**Duration of epilepsy(years)**	6	23	41	4	6
**Follow up duration^2^(years)**	4	8	5	1	2
**Psychiatric comorbidities**	Depression	Anxiety, Depression	N/A	Anxiety, Depression Suicidal ideation	PANS^25^
**Psychiatric treatment(Y/N)**	Y	Y	N	Y	N
**PNES(Y/N)**	Y	Y	N	Y	N
**ASM trial before RNS**	CLB, VPA, CNB, ZNS	LCM, LTG, LEV, CNB, CLB, CZP	PB, CBZ, OXC	PB, LCM, LEV, PER, CZP, TPM	CLB, OXC, PB, BRV
**ASM at last follow up**	CNB, ZNS, PER, LCM, CBD	LCM, LTG, CNB, CLB, CZP	CNB, LEV, LTG, LCM, CLB, VPA, OXC, CZP	PB, LCM, LEV, PER, CZP, TPM, CBD	BRV, LTG, ESL
**RNS implant location**	R lateral temporal lobe and L amygdala-hippocampus	b/l amygdala-hippocampus	b/l amygdala-hippocampus	b/l amygdala-hippocampus	L angular gyrus and L CM
**RNS stimulation parameters (frequency, burst duration, current, pulse width, charge density)**	111 Hz, 5000 ms, 5.9 mA, 160 µs, 3 µC/cm^2^	111 Hz, 5000 ms, 5.5 mA, 160 µs, 1.3 µC/cm^2^	200 Hz, 5000 ms, 2.1 mA, 320 µs, 2.1 µC/cm^2^	200 Hz, 5000 ms, 3 mA, 320 µs, 4.1 µC/cm^2^	200 Hz, 5000 ms, 2 mA, 320 µs, 2.7 µC/cm^2^
**Baseline episodes/day^3^**	673.3	1483.7	1894	83	1791.7
**Current episodes/day^4^**	456.7	958	768.3	149	1157
**DBS stimulation site and parameters (frequency, pulse width, current)**	L CM, R ANT; 145 Hz, 90 µs, 2.5 mA	b/l ANT; 145 Hz, 90 µs, 3.0 mA	b/l ANT; 145 Hz, 90 µs, 4.0 mA	b/l pulvinar; 145 Hz, 90 µs, 1 mA	N/A
**Seizure Reduction(%)^5^**	60	50	>80	50	>60–70

^a^Patient's age at the time of implant; ^2^Follow up duration: time from implantation to most recent follow up for all neurostimulation modalities; ^3^Defined as the average of the first three RNS interrogation data points available; ^4^Defined as the average of the three most recent RNS interrogation data points available; ^5^Refers to overall seizure reduction with all neurostimulation interventions.

ANT: anterior nucleus of the thalamus, ASM: anti-seizure medications, b/l: bilateral, BRV: brivaracetam, CBD: cannabidiol, CBZ: carbamazepine, CLB: clobazam, CM: centromedian nucleus of the thalamus, CNB: cenobamate, CZP: clonazepam, ESL: eslicarbazepine, F: female, FIAS: focal seizures with impairment of awareness, GTCS: generalized tonic-clonic seizure, L: left, LCM: lacosamide, LEV: levetiracetam, LTG: lamotrigine, M: male, N: no, N/A: non-applicable, OXC: oxcarbazepine, PANS: pediatric acute-onset neuropsychiatric syndrome, PB: phenobarbital, PER: perampanel, PNES: psychological non-epileptic seizures, R: right, TPM: topiramate, VPA: valproic acid, Y: yes, ZNS: zonisamide.

A 31-year-old right-handed male presented with intractable bi-temporal lobe epilepsy following encephalitis. The patient had a history of NORSE (new-onset refractory status epilepticus) and remained in a medically induced coma for several weeks following an encephalitis-like illness in October 2017. Seizures continued after his initial critical illness and remained refractory to medications. He experienced 6–10 seizures weekly, which proved resistant to multiple ASMs and a trial of immunomodulatory agents including PLEX, IVIG, cyclophosphamide, and rituximab. Patient also underwent right anterior temporal lobectomy in 2019 at an outside institution, most likely since his initial EEG workup revealed right-sided focal temporal seizure onset. Additionally, he exhibited symptoms of depression, significant memory decline, and episodes of psychosis and hypersexuality.

At first, the patient underwent RNS implantation at a different institution in 2019, specifically targeting the right lateral temporal lobe and left amygdala-hippocampus. At 18 months post-implantation, the therapy led to a 30 % reduction in seizure frequency. Following a video EEG investigation at our institution, studies confirmed two distinct seizure types: a) right arm and face tonic seizures that evolved to bilateral tonic-clonic activity and b) right temporal onset seizures with focal impaired awareness (FIAS). Multifocal partial epilepsy was suspected based on EEG. Intracranial monitoring with sEEG was not further pursued due this suspicion. Due to the characterization of epilepsy foci as well as RNS failure, DBS was a logical next step.

Thalamic targets for DBS were determined by a team of epileptologists, neurosurgeon, neuropsychologist, and a radiologist. Due to anatomic connectivity, targeting the centromedian(CM) nucleus seemed more reasonable in treating generalized and multifocal seizures, while ANT was deemed more appropriate for focal seizures^8^. Therefore, the motor seizures were addressed by targeting CM, while FIAS seizures were addressed through ANT target. DBS targeting right ANT and left CM was subsequently proposed and placed in 2022.

Initially, the DBS settings consisted of continuous stimulation at 145 Hz, 2 mA, and 90 mS. However, due to reported sleep disturbances and worsening mood, the settings were adjusted to continuous stimulation at 10 Hz with a pulse width of 130 mS and a current of 3.3 mA. This intervention led to an overall 60 % reduction in seizures, with resolution of seizure clusters. Notably, RNS ECoG played a crucial role in identifying the underlying cause of psychosis linked to increased detection of electrographic seizure-like activity (called as long episodes) overnight. This correlation was determined based on correlation of RNS ECoG data with clinical report of onset of psychotic symptoms, including paranoia, auditory hallucinations, and delusions. The episodes were effectively managed acutely with lorazepam treatment. In long term follow-up, DBS was the primary device adjusted, whereas RNS settings remained constant. Overall, the patient self-reported improved sleep after the DBS stimulation frequency was adjusted to 10 Hz.

Prior to neuromodulation, the patient’s medication regimen consisted of clobazam, valproic acid, cenobomate, and zonisamide. Throughout the follow up period, medications were adjusted. Notably, perampanel, lacosamide and cannabidiol were added to the regimen, while valproic acid and clobazam were discontinued.

Case #2(combined VNS, RNS, and DBS neuromodulation)

#2**-** A 45-year-old right-handed male developed intractable multifocal epilepsy following viral encephalitis, which initially manifested as febrile illness and generalized convulsive status epilepticus when he was 24 years old. After a flu-like illness at an outside institution, patient presented with a combination of encephalopathy and seizures, leading to a diagnosis of encephalitis. However, the cause of the encephalitis was not determined. Over time, he began experiencing weekly 4–6 FIAS that proved resistant to more than eight ASMs. Imaging revealed left frontal focal cortical dysplasia. In correlation with this lesion found on imaging, frontal seizure semiology was found clinically. Therefore, awake left frontal lesionectomy was proposed by an expert team. Attempts to control his seizures through VNS and ECoG-guided left frontal resection were unsuccessful. Additionally, he suffered from severe memory loss and developed nonepileptic spells (confirmed by scalp video EEG) alongside his seizures**.**

**Fig. 1:**
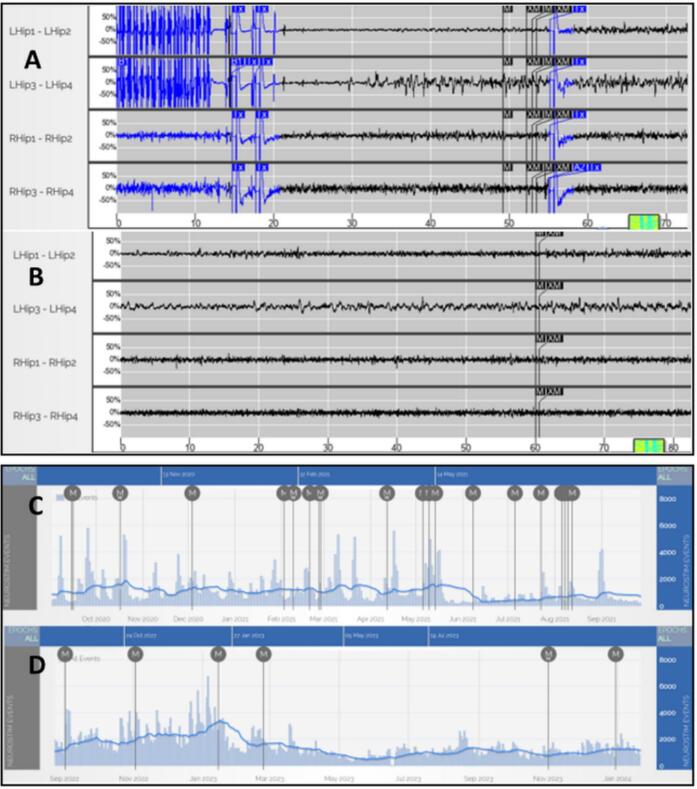
RNS device recordings showing seizures (A and C) and psychogenic nonepileptic seizure (PNES) (B and D): Two examples of magnet swipe ECoGs: ECoG A shows seizure termination and postictal state. The patient reported increased anxiety and subsequent loss of awareness during the seizure. ECoG B shows the PNES event with no ictal EEG changes and the event was characterized by anxiety without loss of awareness. (C) RNS event tracker shows frequent magnet swipe events. Most of these events were PNES. This warranted cognitive behavioral therapy. (D) RNS tracker post-therapy shows a marked decrease in PNES episodes as well as magnet swipes. RNS-Responsive Neurostimulation; ECoG-electrocorticography.

Video EEG confirmed bi-temporal lobe epilepsy. Initially, the patient underwent RNS targeting the bilateral amygdala-hippocampus to address his condition. Six years post-RNS therapy, there was a 40 % reduction in seizures. Furthermore, the RNS facilitated the refinement of Cognitive Behavioral Therapy (CBT) for nonepileptic spells, aided by RNS ECoG, which assisted in distinguishing epileptic events from nonepileptic ones (Fig. 1). Characterization by scalp EEG revealing bi-temporal lobe epilepsy supported DBS as the next step in treatment since a relatively equal proportion of seizures arose from the left and right side [9]. This lack of strong asymmetry resulted in the decision to not pursue further intracranial monitoring and move forward with DBS.

**Fig. 2:**
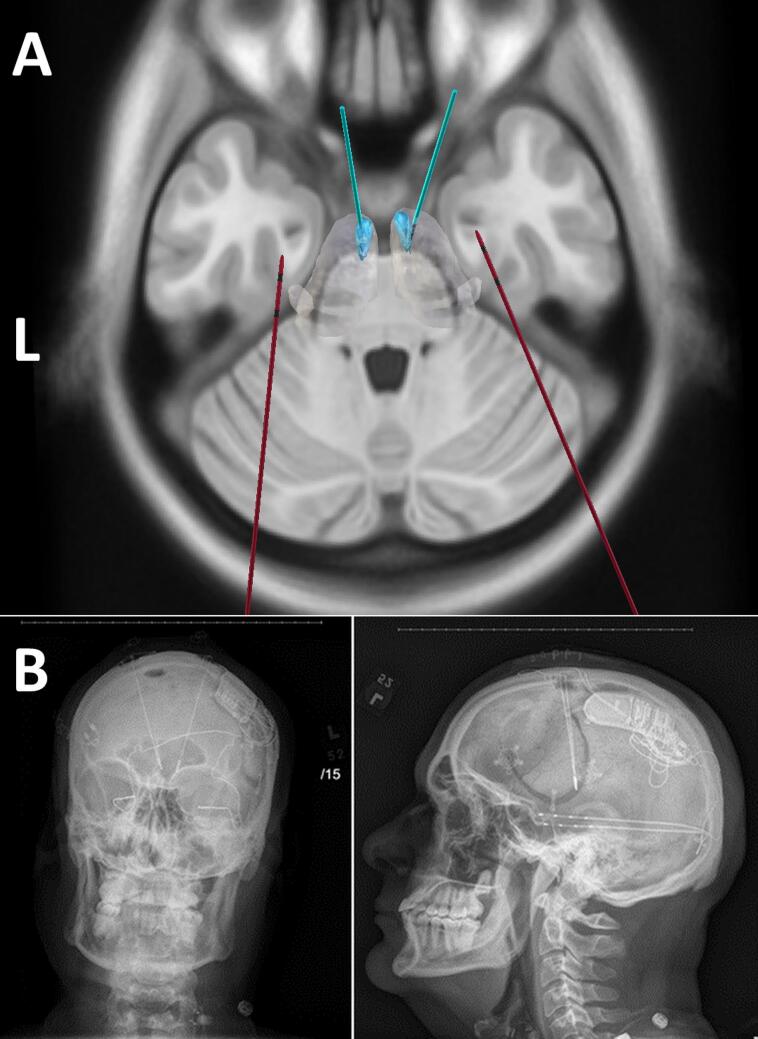
(A) Multimodal neuromodulation implant: An example of a patient who had received both anterior nucleus (ANT) of thalamus deep brain stimulation (DBS) as well as hippocampal responsive neurostimulation (RNS) and the vagal nerve stimulator (VNS): The accuracy of implantation was determined by co-registering post-implant CT into pre-operative MRI images using non-linear transform in advanced normalization toolbox. Thalamus Optimized Multi Atlas Segmentation (THOMAS) segmentation algorithm was used to generate patient specific thalamic nucleic segmentation (blue regions represent the bilateral ANT nuclei, the gray blob represents the thalamic boundary). DBS electrodes (cyan) were localized to the ANT and the bilateral RNS electrodes (red) were localized to the hippocampi. (B) Anteroposterior and lateral fluoroscopy images showing the neurostimulator placements for vagus nerve stimulation (VNS), responsive neurostimulation (RNS), and deep brain stimulation (DBS). VNS lead with terminal electrodes placed adjacent to a segment of the left vagus nerve (cuff on vagus nerve). (For interpretation of the references to colour in this figure legend, the reader is referred to the web version of this article.)

In May 2022, the patient underwent bilateral Anterior Nucleus of the Thalamus Deep Brain Stimulation (ANT-DBS) implantation (Fig. 2 A and B). This target was determined by the team due to efficacy in FIAS [8]. By 2023, RNS and DBS interrogation indicated a reasonable > 65 % percentage seizure reduction from 2021 seizure burden. RNS was also adjusted during this time period.

During the follow up period, levetiracetam was discontinued while the other five medications remained the same dose.

Case #3(combined VNS, RNS, and DBS neuromodulation)

# 3**-** A 53-year-old left-handed female developed bitemporal epilepsy following catastrophic encephalitis of unknown origin when she was 12 years old. Her initial presentation included nonconvulsive status epilepticus, and MRI brain scans revealed hippocampal sclerosis, bilateral temporal regional, and global atrophy. Encephalitis was diagnosed on strong clinical suspicion after rule out of other conditions and correlation with presentation. Patient had no history of head trauma, cerebrovascular injury, febrile seizures or family history of any seizure disorder. Despite undergoing trials with more than 9 ASMs and VNS, she continued to experience weekly temporal onset focal aware seizures (FAS) and FIAS. Additionally, she had 2–4 bilateral focal bilateral tonic clonic (FBTC) seizures annually. Scalp EEG revealed focal epilepsy with secondary generalization. However, seizure focus was not well established. Therefore, patient underwent stereoelectroencephalography (sEEG), on which bilateral mesial temporal lobe epilepsy was confirmed with an equal proportion of seizures arising from right and left temporal lobe. Due to the characterization of two seizure foci, bilateral amygdala-hippocampal RNS was preferred over DBS [9].

In an effort to manage her condition, the patient underwent bilateral amygdala-hippocampal RNS, which resulted in a 60 % reduction in seizures. However, she still experienced monthly FIAS and FAS. After four years of treatment with RNS and desire for additional seizure control, the decision was made to pursue DBS. Bilateral ANT was proposed as a target due to the patient’s focal seizure types [8]. Consequently, in 2022, the patient opted for bilateral ANT-DBS, which effectively stopped her FBTC seizures. During her most recent clinic visit, she reported experiencing only one FIAS seizure per month, representing an > 80 % reduction in her overall seizure frequency.

Prior to neuromodulation, the patient’s medication regimen consisted of phenobarbital, carbamazepine, and oxcarbazepine. However, medications were adjusted throughout the followup period, and the patient was on eight seizure medications at the end of followup listed in Table 1. She continued with oxcarbazepine from the initial regimen. Increasing the number of seizure medications from 3 to 8 is a significant confounding factor when evaluating the efficacy of her neuromodulation.

Case #4(combined VNS, RNS, and DBS neuromodulation)

#4- A 25-year-old right-handed female developed intractable bi-limbic epilepsy as a consequence of febrile infection-related epilepsy syndrome (FIRES). To address her condition, she underwent pharmacologically induced coma treatment for several weeks. Brain biopsy results confirmed encephalitis, which was subsequently managed with intravenous immunoglobulins and methylprednisolone.

Following her hospitalization, she experienced a significant cognitive decline, profound memory loss, and depression, leading to a suicide attempt. Alongside these challenges, she also developed nonepileptic spells, in addition to experiencing 10–12 FIAS weekly. Despite an attempt with VNS to control her seizures, it was deactivated due to major discomfort. Bilateral temporal lobe epilepsy was discovered on EEG monitoring. Consideration of both imaging findings and EEG monitoring supported two seizure foci. Therefore, intracranial monitoring was not required to move forward with the decision to pursue RNS due to its efficacy in epilepsy with one or two seizure foci[9].

A bilateral amygdala-hippocampal RNS device was implanted with parameters set at 200 Hz, 5000 ms, and 3 mA to better manage her condition. Two years post-intervention resulted in a reduction of over 40 % in observed clinical seizures. The family was concerned about SUDEP, so DBS was offered. When selecting a target, the patient's extensive psychiatric history, which included a suicide attempt, ruled out the possibility of ANT-DBS. Previous studies have demonstrated the efficacy of pulvinar DBS bitemporal refractory epilepsy[8], [9], [10]. In addition, other targets such as subthalamic nucleus and CM are considered more appropriate for motor seizures and generalized seizures, respectively[8]. Therefore, bilateral medial pulvinar DBS was offered to the patient. The patient had DBS for two months, and hence, the long-term outcome is not available. At most recent follow up in January 2024, seizure episodes decreased by 50 %, from once weekly to once every two weeks. In addition, mood remained stable.

In this patient, the medication regimen prior to neurostimulation consisted of six medications listed in Table 1. By the end of follow up, the six medications were kept the same and only cannabidiol was added to the regimen.

Case #5 (Combined thalamocortical RNS)

At the age of 7, a 13-year-old right-handed male developed multifocal epilepsy following an episode of FIRES. After recovering from FIRES, his MRI brain scan revealed left-sided hippocampal signal changes, with more pronounced abnormalities on the left side but not definite sclerosis, accompanied by temporal lobe atrophy. Due to presentation of FIRES and rule out of other conditions, encephalitis emerged as a likely diagnosis.

Patient initially presented to outside institution with available records. Scalp EEG did not provide the required clarity to characterize seizure foci since mostly subclinical seizures were recorded. The results suggested clinical and electrographic seizures arising from left temporoparietal region. On repeat scalp EEG, multifocal activity was found. To further investigate his condition, the patient underwent bilateral stereo EEG, which confirmed onset of seizures from left angular gyrus, left posterior mesial frontal lobe, and left middle temporal gyrus anteriorly. Results were suggestive of focal cortical dysfunction in both hemispheres. Traditionally, RNS to CM nucleus has been preferred for prominent motor or frontal involvement while RNS to ANT has been used in limbic involvement [11]. EEG revealed both right and left focal motor seizures. sEEG results revealed four different types of seizure events. The most severe and common event in this patient had consistent electrographic onset from the left angular gyrus confirmed by sEEG. The second and third types of seizures arose from the left anterior temporal region, in the middle temporal gyrus and from the left posterior frontal region near the midline, respectively. The last seizure type arose from the right hemisphere. The left centromedian nucleus was sampled along with the left angular gyrus through cortical stimulation due to anatomic connectivity and sEEG results, respectively. This information also aided in selecting the appropriate targets. Subsequently, RNS targeting the left angular gyrus and left CM thalamus was recommended by an expert team at an outside hospital. After sEEG and RNS were initially placed at outside institution, patient subsequently followed up at our institution, where RNS was adjusted. Two years post-implantation, the patient's family reported a reasonable > 60–70 % reduction in both seizure frequency and epileptiform activities.

Prior to RNS, the patient was on clobazam, phenobarbital, oxcarbazepine, and brivaracetam. By the end of follow up, brivaracetam was kept on the medication regimen, but lamotrigine and eslicarbazepine were added. In turn, clobazam, phenobarbital, and oxcarbazepine were discontinued. Overall, the patient’s regimen decreased from four to three medications during the follow up period.

## 4 Discussion

Postencephalitic epilepsies pose a complex challenge, primarily due to the presence of significant neuropsychiatric and cognitive comorbidities, in addition to frequent drug-resistant seizures [12].

In this study, we explore the use of multimodal neuromodulation therapies that target multiple nodes within the thalamocortical network. Notably, four of the patients in our cohort experienced a seizure reduction of at least 50 %, indicating the potential efficacy of these treatments in encephalitis patients.

Previous research has delved into the relationship between anti-inflammatory effects and VNS in epilepsy management [13], [14], [15]. Similarly, another study has highlighted the anti-inflammatory properties of DBS in the context of status epilepticus [16]. These findings suggest that neurostimulation may hold promise as an effective approach to managing encephalitis-related epilepsy. Consequently, prior investigations have explored the use of DBS, RNS, and VNS in encephalitis patients with chronic epilepsy [17], [18], [19].

For instance, in a case of anti-GAD (glutamic acid decarboxylase) encephalitis treated with DBS, the patient experienced a reduction in seizure frequency, with events decreasing from 90 to 160 per month in the six months before DBS implantation to 30–70 per month afterward [17]. Similarly, a report on RNS for Rasmussen's encephalitis demonstrated a 50 % reduction in seizure intensity, and a study on VNS in anti-GAD encephalitis resulted in a transient 75 % reduction in seizure frequency [18].

Another consideration is the timing of neuromodulation in the patient's disease course. In this case series, two patients suffered from FIRES and one patient suffered from NORSE. Our results support the efficacy of neuromodulation even in the chronic setting. In a recent study of 20 patients with NORSE/FIRES diagnosis, neuromodulation consisting of ECT, VNS, or DBS resolved status epilepticus in 18 patients [20]. In addition, results suggested that earlier administration of neuromodulation in the acute setting could be more efficacious in resolving status epilepticus [20]. When considering this data, it is possible that the efficacy of this treatment is understated in our series.

However, it's worth noting that there is limited existing literature regarding the use of multiple types of neuromodulation in chronic epilepsy stemming from encephalitis. Highlighting the critical advantage of RNS ECoG in postencephalitis patients is pivotal within the context of this case series. These patients frequently present with concurrent nonepileptic spells and neuropsychiatric symptoms that may be intricately linked to the *peri*-ictal state, encompassing ictal or postictal psychosis, or comorbid psychiatric conditions [21], [22]. The RNS ECoG can help in classifying the events as epileptic or nonepileptic spells. It's important to note that these two categories of symptoms necessitate different treatment approaches, with antiseizure medications addressing the former and various psychotropic medications addressing the latter [23].

In this case series, the application of RNS ECoG emerges as a valuable tool that assists in elucidating the nature of these events, distinguishing between epileptic and nonepileptic phenomena, and tailoring therapy accordingly. In correlation with clinical data, RNS ECoG can be useful as a confirmatory study in differentiating these presentations. In addition, the utility of RNS ECoG in detecting seizure activity is only increasing with recent advances in machine learning algorithms [24]. Machine learning advances could enable the RNS system to more accurately detect epileptic events with higher confidence, making it adaptive and continuously refining its ability to distinguish epileptic event types from non-epileptic events as more data is collected with ECoG and clinically correlated. Therefore, we encourage further research and consideration in the use of RNS ECoG data in this manner. Multiple subjects in the case series exemplify the utility of RNS ECoG in this regard, further underscoring its significance in optimizing the management of postencephalitis patients with multifaceted neuropsychiatric presentations.

In conclusion, employing a combination of neuromodulation techniques for the treatment of epilepsy in encephalitis patients can yield positive outcomes. Nevertheless, further research is warranted to better understand the predictors of response to neuromodulation therapies in a broader context. A prospective confirmatory study with a larger sample size is needed. The impact of each individual device within poly-therapy should be analyzed using internal controls to prospectively evaluate their effects.

Additionally, there is a pressing need for more comprehensive investigations into the application of neuromodulation as a therapeutic approach for chronic epilepsy arising from encephalitis, thus paving the way for enhanced care and improved quality of life for these patients.

## 5 Ethical publication statement

We confirm that we have read the Journal's position on issues involved in ethical publication and affirm that this report is consistent with those guidelines.

## CRediT authorship contribution statement

**Subhiksha Srinivasan:** Writing – review & editing, Visualization, Validation. **Surya Suresh:** Writing – review & editing, Visualization, Validation. **Ganne Chaitanya:** Writing – review & editing, Methodology, Data curation. **Manoj Saranathan:** Software, Data curation. **Nitin Tandon:** Resources, Conceptualization. **Sandipan Pati:** Writing – original draft, Visualization, Validation, Supervision, Project administration.

## Declaration of competing interest

The authors declare that they have no known competing financial interests or personal relationships that could have appeared to influence the work reported in this paper.
